# Progression of valvular heart disease in dialysis patients: how to stop it?

**DOI:** 10.1590/2175-8239-JBN-2024-E005en

**Published:** 2024-04-08

**Authors:** Eduardo Leal Adam

**Affiliations:** 1 Universidade Federal do Paraná, Hospital de Clínicas, Curitiba, PR, Brazil.

Patients with end-stage renal disease (ESRD), especially those receiving renal replacement therapy, are at increased risk of cardiovascular disease. Although sudden death, heart failure, and coronary artery disease are the most frequent presentations of cardiac disease in this population, valvular heart disease (VHD) has recently emerged as a common comorbidity in dialysis patients^1^. Importantly, valvular involvement is associated with an adverse prognosis in patients with ESRD^2^.

Risk factors for VHD in the general non-dialysis population are also present in patients with ESRD, namely hypertension, diabetes, lipid disorders, and advanced age^3^. Nevertheless, risk factors specific to patients in dialysis render VHD more frequent in this scenario than in patients with normal renal function. Abnormal metabolism of calcium and phosphorus, hyperparathyroidism, low-grade chronic inflammation, malnutrition, altered hemodynamics, and increased shear stress accelerate the development of valvular calcification and degeneration, with progression rates of valve dysfunction greater than those observed in the general population^4^.

The paper published by Tompson et al.^5^ presents the results of an observational study performed at a single center in Brazil. Two hundred ninety-one patients receiving renal replacement therapy were screened for VHD using transthoracic echocardiography. There was a high prevalence of mitral (82.5%) and aortic (65.6%) valve involvement in this cohort. Progression of at least one degree of valve dysfunction (none to mild, mild to moderate, or moderate to severe) was observed in 36.4% of patients during follow-up. Longer time in dialysis was significantly associated with the presence of mitral and tricuspid valve disease in univariate analyses, but that association was no longer significant after adjustment for age, diabetes status, hyperparathyroidism, and type of renal replacement therapy. Hyperparathyroidism was the only variable significantly associated with mitral valve disease after multivariate analysis.

The authors bring significant contributions to the field of VHD in patients with ESRD, an underrepresented area in medical research, reinforcing the high prevalence and high rate of progression of valve disease in this population. Previous studies have shown progression rates of aortic valve stenosis to be more than two-fold compared to patients without renal disease^4^. Also, patients in dialysis undergoing surgery for valve replacement are usually considered at risk for accelerated calcification and dysfunction of biological prosthesis^6^. In the study of Tompson et al.^5^, two of the four patients receiving tissue valves developed prosthesis calcification during follow-up.

Limitations of the study are: (i) although valvular involvement was common, the degree of severity of valve dysfunction was not reported; (ii) it is not clear if all patients underwent serial echocardiographic examinations, in whom repeat exams where performed, and the time interval between them was not standardized, which could lead to biases in the analysis of disease progression; (iii) due to its retrospective design, missing data in medical charts may have influenced the results of the study, as stated by the authors; iv) echocardiographic evaluation of valvular dysfunction depends on cardiac preload and afterload; therefore, serial echocardiographic examinations should ideally be performed after dialysis sessions to avoid false disease progression or regression due to imbalances in hemodynamic conditions between exams.

The molecular pathways of valve disease development and progression are still poorly recognized in patients with ESRD, and a deeper understanding of its unique pathophysiology is necessary to develop effective ways to halt its occurrence. A randomized trial with desonumab and alendornic acid in patients with calcific aortic stenosis and normal renal function showed no benefit of these therapies for prevention of progressive valvular dysfunction^7^. Also, trials with statins and ezetimibe have shown no benefit in decreasing aortic valve deterioration^8^. However, a small clinical trial of patients with ESRD using sevelamer as an alternative to calcium-based phosphorus binders have shown slower rates of valvular and vascular calcification in the sevelamer group, as evaluated by cardiac computed tomography^9^. Although the trial was not powered for the assessment of hard outcomes, this strategy might be considered on an individual basis for prevention of valvular deterioration in patients with ESRD.

In conclusion, the study of Tompson et al.^5^ adds to the epidemiological data showing high prevalence and progression of VHD in patients with ESRD. None of the classic cardiovascular risk factors or time in dialysis were associated with VHD, but attention must be taken for the adequate treatment of hyperparathyroidism, considering its observed association with mitral valve disease.
